# Respiratory Syncytial Virus: A Comprehensive Review of Transmission, Pathophysiology, and Manifestation

**DOI:** 10.7759/cureus.36342

**Published:** 2023-03-18

**Authors:** Jasndeep Kaler, Azhar Hussain, Kishan Patel, Tamara Hernandez, Sidhartha Ray

**Affiliations:** 1 Medicine, Windsor University School of Medicine, Cayon, KNA; 2 Pharmacology and Therapeutics, Touro College of Pharmacy, New York, USA; 3 Pediatric Pharmacist, Touro College of Pharmacy, New York, USA; 4 Pharmaceutical and Biomedical Sciences, Touro College of Pharmacy, New York, USA

**Keywords:** pathophysiology of rsv, paramyxoviridae, paramyxovirus, pneumonia, respiratory syncytial virus

## Abstract

With an increasing global incidence in children younger than the age of five, respiratory syncytial virus (RSV) is one of the most common viral respiratory infections worldwide. Despite the increasing number of cases among infants and young children, RSV can infect any age group; however, some individuals are more high risk than others. Premature infants, young children, elderly, and immunocompromised individuals are the most likely to suffer a more severe presentation of RSV in comparison to healthy adults. RSV is transmitted through respiratory droplets via direct contact with an infected individual or with contaminated surfaces. The viral genome of RSV consists of 11 proteins. Out of these 11, two proteins allow for the attachment of the virus to the respiratory epithelial cells and fusion with host cells. Upon fusion, the viral material transfers to the host cell, where viral replication occurs. It is important to acknowledge that an individual is considered infectious and can transmit the virus even before the symptomatic presentation of RSV begins. As long as the individual is shedding the virus, he or she is considered infectious. The length of viral shedding also differs depending on the severity of the infection, who is infected, and the underlying immune status of an individual. Currently, there is no definitive treatment for RSV; however, supportive therapy is considered the mainstay treatment. Some pharmaceutical treatments such as ribavirin have been FDA-approved; however, the administration is typically limited to children and infants. Palivizumab is also administered as an immune prophylaxis; however, both therapies are constantly at the end of a cost-effective debate due to their extensively expensive nature and questionable adverse effect profiles. Supportive therapy includes hydration, supplemental oxygen, and mechanical ventilation in hospitalized cases; however, most RSV cases can be treated as outpatient cases. Prevention techniques such as hand washing and maintaining social distancing are imperative to minimize the transmission of the virus as much as remotely possible.

## Introduction and background

Respiratory syncytial virus (RSV) infection has an estimated global incidence of over 30 million cases in children aged five years or less, with hospitalization occurring in less than 10% [1]. According to the Centers for Disease Control and Prevention (CDC), RSV transmission generally starts during fall and peaks in winter [2]. Hospitals feel the burden of an all-time high presentation of RSV cases in recent years. However, the seasonal nature of RSV transmission does vary globally. In regions with milder temperatures, RSV rates increase during late autumn, winter, and/or spring, whereas tropical and arctic climates observe RSV cases year-round [1]. In more tropical regions, the seasonal nature of RSV has been noted with a decrease in temperature and an increase in rainfall. In contrast, annual outbreaks are prevalent in more tropical climates during the warm and rainy seasons [1,3]. It has been postulated that the correlation of an increase in RSV cases with declining temperatures has been associated with increased indoor crowding that can further viral transmission. Lower temperatures increase viral stability and host susceptibility or activation of the dormant virus [4]. Recent studies have also reported an association between COVID-19 and RSV seasonality changes. Along with individual behavioral changes and viral interference, control measures against SARS-CoV-2 could have delayed the RSV outbreak in the summer of 2021 [1-4].

Historically, RSV has been one of the most common causes of lower respiratory infections in infants and young children. During the first year of life, maternal immunoglobulins do not adequately protect infants from RSV. Thus, approximately 80% of lower respiratory tract infections in children younger than one year of age are due to RSV, with a peak incidence occurring at two to three months of age [4]. However, recently, RSV has been noted as a common pathogen amongst the elderly and immunocompromised patients as well. The bimodal presentation of RSV causes a majority of the cases to be concentrated amongst the pediatric and geriatric populations.

Depending on the group of individuals infected by RSV, the symptomatic presentation will differ. Healthy adults may be more asymptomatic than the elderly, immunocompromised, or other high-risk patients. Furthermore, individuals with other comorbidities or chronic health conditions tend to be at an increased risk of RSV infection. Some of the risk factors for progression to viral pneumonia and other complications include Down's syndrome, compromised immunity (including patients receiving chemotherapy or chronic immunosuppression for connective tissue disease/vasculitis), underlying lung disease (especially asthma) or heart disease, old age, frailty, living in a long-term care facility, and living at high altitudes [3]. Environmental factors can also influence the development of RSV disease among individuals. Tobacco smoke, air pollution, and indoor crowding are some of the environmental exposures that elevate the risk of RSV infestation [1].

**Figure 1:**
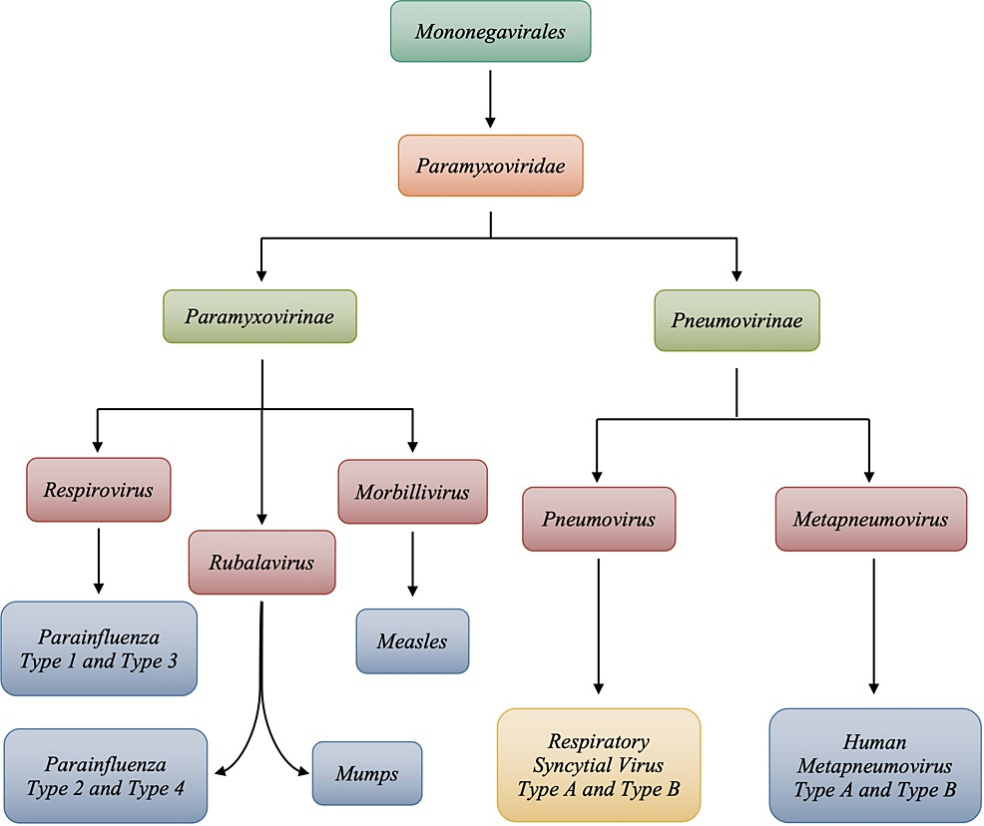
Lineage of Paramyxoviridae and Respiratory Syncytial Virus Image credits: Jasndeep Kaler, Azhar Hussain, Kishan Patel, Tamara Hernandez, Sidhartha Ray

Mononegavirales is a non-segmented single-stranded negative-sense RNA virus [1,4]. Some of the most pathogenic viruses that have been derived from the mononegavirales order are known as the Ebola virus, rabies virus, measles, and RSV. From the order mononegavirales, RSV is from the paramyxoviridae lineage. The paramyxoviridae family branches into two subfamilies: paramyxoviridae and pneumovirinae. As depicted in Figure 1, the RSV is part of the pneumovirus genus that belongs to the pneumovirinae subfamily. The other genus under the pneumovirinae classification is the metapneumovirus genus. The metapneumovirus genus gives rise to the human metapneumovirus type A and type B (hMPV). hMPV is also a significant pathogen in children. Symptoms of RSV and hMPV infection are very similar, and often distinguishing between the two is difficult; however, there are differences in viral proteins that allow for the differentiation of the two infectious pathogens. The family paramyxoviridae also gives branches to the paramyxovirinae subfamily, which gives rise to viruses such as parainfluenza, measles, and mumps. RSV is known to be the most complex member of the family pneumovirinae due to its extensive number of genes and proteins [4].

RSV is a negative sense, single-stranded, enveloped RNA genome composed of approximately 15,222 nucleotides and 10 genes that encode 11 proteins [1,5-6]. RSV has a non-segmented genome, so unlike influenza, it cannot reassort genome segments and thus undergo antigenic shifts that can potentially cause large-scale pandemics [1]. However, as with other RNA viruses, RSV has a quite mutable genome by its dependence on an RNA polymerase that lacks the capacity for RNA proofreading and editing [6]. Due to this inherent characteristic or property, variations of RSV exist despite antigenic variability. These variations of RSV have been indicated in Figure 1.

**Figure 2:**
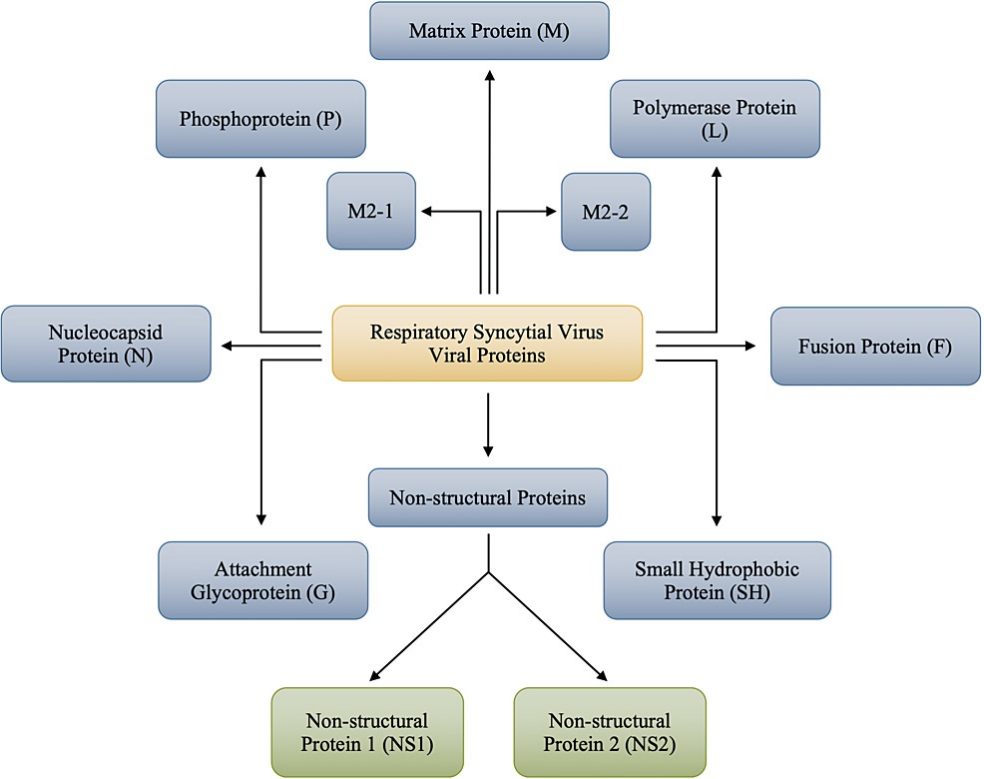
Viral Proteins of Respiratory Syncytial Virus (RSV) Image credits: Jasndeep Kaler, Azhar Hussain, Kishan Patel, Tamara Hernandez, Sidhartha Ray

The RSV is classified into two specific and distinct subgroups: type A (RSVA) and type B (RSVB). This subgrouping is based on antigenic variability. Antigenic variability refers to differences in proteins amongst the causative agent that, in this case, allows RSV to evade the host immune response leading to the possibility of reinfections. Additional antigenic variability occurs within RSVA and RSVB. It plays a significant role in RSV pathogenicity and immune evasion, due to its extensive antigenic and genetic diversity found in the attachment glycoprotein C [1,5]. In contrast, glycoprotein F, responsible for virion membrane fusion, is highly conserved amongst RSVA and RSVB strains and thus may become the focus of future pharmaceutical treatments for RSV [6]. Immunological diversion by an RSV type during one season may render the population more susceptible to the other type in the following season [7]. The 10 genes encoded by the RSV genome translate 11 viral proteins, as shown in Figure 2.

Each of the viral proteins studied concerning RSV plays a pivotal role in either the attachment and infectivity of the pathogen or its replication and transcriptive properties. Figure 2 outlines two non-structural proteins that are called non-structural protein 1 (NS1) and non-structural protein 2 (NS2). Since NS1 and NS2 are referred to as non-structural proteins, it can be ascertained that both are not present in the mature virion [8]. These non-structural proteins’ presence distinguishes RSV from the other pathogens under the mononegavirales order, including the closely related hMPV [8-9].

Furthermore, NS1 and NS2 have evolved into proteins that have acquired multiple ways to suppress type I interferon (IFN) production and signaling [8]. Like other RNA viruses, RSV triggers the toll-like receptors (TLRs) and the retinoic acid-inducible gene I (RIG-I) pathways upon infection to initiate a signal cascade that leads to the production of type 1 IFN [10]. The predominant IFN that is induced by the nasal epithelium in response to an RSV infection is known as interferon-gamma (IFN-γ) [8-9]. The suppression of IFN production and signaling is one of the processes through which the viral proteins of RSV can overcome the host’s innate immune system. IFN-γ is a type III IFN, which is implicated as the primary IFN to protect the airway epithelial cells against any respiratory infection [10]. Furthermore, the interference with type I IFN response by NS1 and NS2 proteins blocks dendritic cell (DCs) maturation. This effect in DCs could interfere with their capacity to activate T cells [11-13]. Suppression of the IFN production due to the presence of NS1 and NS2 proteins causes the airway epithelial cells to be more susceptible to increased damage at the hands of any type of respiratory infection. In addition, human bronchial epithelial cells expressing NS1 and NS2 proteins decrease T cell polarization toward Th1, Th2, and Th17 phenotypes by the NS1 protein and Th2 and Th17 polarization by the NS2 protein [5,11-13]. Both these non-structural proteins are crucial virulence factors that directly impact the host's immune response. 

While NS1 and NS2 inhibit the host’s innate immune system, other viral proteins of RSV mentioned in Figure 2 will further increase the likelihood of infectivity. The nucleocapsid protein (N), along with the phosphoprotein (P), transcription factor M2, and large RNA-dependent RNA-polymerase (L) are all found in an inner nucleocapsid that wraps the genomic RNA [11]. NS1 and NS2 are found only in infected cells but not in virions [12]. Matrix (M) protein is a non-glycosylated protein located underneath the envelope, which has been discovered to be involved in the assembly of viral particles [11-12]. The G glycoprotein (G) mediates viral attachment, and the fusion (F) protein mediates viral penetration and syncytium formation [12]. Furthermore, the small hydrophobic (SH) protein was recently postulated to be a viroporin-like ion channel, interfering with the normal function of the host cell, allowing infection to be possible [10-12]. Proteins N, L, P, and the viral RNA collectively constitute the viral replication (VR) complex.

As a template for RNA synthesis, the 391 amino acid N protein binds tightly to the genome and antigenome to form helical nucleocapsids [5]. N protein binds to the RNA genome in a way that prevents the RNA genome to form a double-stranded structure [13]. Preventing the RNA genome to form its double-stranded RNA structure allows for viral replication and transcription to occur much easier [8,13]. The N protein is also involved in sequestering the innate immune system’s signaling proteins into inclusion bodies (IB) [8,10]. The N protein is sequestered within cytoplasmic IBs, where it interacts with the M1, P, and L proteins [13]. These IBs are considered to be the sites of viral replication and transcription, where all viral proteins of the VR complex are concentrated [8]. N protein has another important function in inhibiting interferon signaling response and function in cell stress response, such as inducing apoptosis [8,10]. Furthermore, it has a role in antagonizing the host’s innate immunity. Following the first hours of an RSV infection, the N protein has been shown to associate within the IBs with melanoma differentiation-associated gene 5 (MDA5) and mitochondrial antiviral signaling (MAVS), which contribute to the innate immune response [13]. By sequestering these molecules, nucleic acid sensors would have difficulty detecting the viral genome, reducing the effectiveness of IFN [10,13]. Due to a blockage of the interaction between peptide-major histocompatibility complexes (pMHCs) and the T-cell receptor, N protein expression impairs the ability of DCs to activate T cells [13]. This role of the N protein allows for new insights into how the induction of protective T-cell responses is often impaired during an RSV infection [8,13].

Viral P protein is a 241-amino acid that is described as a cofactor of the VR complex and is also an essential protein for the functioning of the L protein [5,13]. The P protein is highly stable and acts as an adapter that binds to N, L, and M2 proteins to mediate interactions in the VR complex [5,13-14]. As a part of the VR complex, the P protein plays a crucial role in RNA transcription and replication. Viral replication and transcription may be hindered without the P protein. The P protein is a major phosphorylated RSV protein containing a phosphate at more than 10-12 sites, with different sites exhibiting differing rates of turnover due to the interplay between cellular kinases and phosphatases [5,14]. The hypothesis that phosphorylation is necessary for RSV replication was confirmed by studies in which P protein phosphorylation was decreased by mutational ablation or the application of a cellular kinase inhibitor [14]. Moreover, the P protein will bind to any free N protein monomers and transport them to antigens, stopping N from self-aggregating or attaching to nonviral RNA [5].

The L protein is 2,165 amino acids in length, and this protein’s principal role is the viral genome’s replication and transcription, regulated and supported by the VR complex [5,13]. Because RSV has a negative-sense genomic RNA, the P protein transcripts the L protein into messenger RNA (mRNA) to express each RSV gene [13]. In this process, the L protein recognizes a promoter region in the 3’ extreme of the negative sense RNA strand and starts the transcription of each gene [5,13]. The L protein then copies the viral genome from a negative sense RNA into a positive sense RNA during replication, resulting in the antigenome. Subsequently, fresh negative sense RNA is created using the antigenome as a template [13]. The newly generated negative sense RNA genomes will then be encapsulated into the generated infective virions.

The M protein, which is 256 amino acids in length, plays a crucial role in virion morphogenesis [5]. Furthermore, the M protein is essential for the replication of the RSV genome as it promotes viral assembly [13]. In the early stages of infection, the M protein is detected in the nucleus, which plays a crucial role in decreasing the transcriptional activity of the host cells’ genes [5,13,15]. Later in the infection, the M protein is noted to also be associated with the cytoplasmic viral IBs [5]. An additional role of the M protein is to arrest the G1 and G2/M phases of the cell cycle in human bronchial epithelial cells [15]. These actions of M protein, which are p53 dependent, increase RSV replication [5,15]. Furthermore, the M protein has been associated with the direct maturation of the viral filaments. The viral filaments are thought to be precursors for the infectious virus to attach to host cells [5]. Mitra et al. suggest that the M protein may not be required to initiate the formation of viral filaments, but in the absence of the M protein, the filaments remain hindered and immature [16]. Other studies show that the M protein is expressed in IBs and interacts with the M2-1 protein to interact with the VR complex [13]. Furthermore, the M protein can also inhibit viral transcription and interacts with the G and F proteins to signal for the assembly of the virions [5,13].

The M2-1 protein is involved in the transcription process as a part of the VR complex. It acts as an elongation factor promoting the transcription of all RSV genes, aiding the L protein to proceed with the transcription of the viral genes [13]. The M2-1 protein is 194 amino acids in length [5]. M2-1, which exists in either a phosphorylated or unphosphorylated form in infected cells, is an RNA-binding protein that interacts with other components of the VR complex [17]. As RNAses disrupt the binding between the M2-1 protein and the N protein, this interaction is particularly mediated by interactions with the viral RNA [13]. Another function of the M2-1 protein is activating the nuclear factor-kappa β (NF-κβ) [13,18]. NF-κβ is one of the pivotal regulators of pro-inflammatory gene expression and induces the transcription of pro-inflammatory cytokines, chemokines, and adhesion molecules, amongst others [18]. For the M2-1 protein to fully exert its effect as an elongation factor, it must be in the form of tetramers. Without the oligomerization, the protein is unable to function correctly, which is supported by using a mutant virus for the M2-1 protein that cannot generate tetramers [13]. Like the other proteins that are part of the VR complex, the M2-1 protein also plays a critical role in the regulation of transcription and replication of the RSV RNA.

Another one of the 11 proteins encoded by the viral genome of RSV is the M2-2 protein. The M2-2 protein is about 88-90 amino acids in length and is expressed in low levels in infective cells [5]. The functionality of the M2-2 protein as a virion component is unknown; however, deletion of M2-2 from recombinant RSV results in a virus that exhibits delayed and reduced RNA replication [5,19-20]. It has been postulated that the M2-2 protein is involved in regulating transcription and replication by the virus polymerase [20]. The overall importance of the M2-2 protein in the RSV viral genome requires further attention and research.

The G protein is one of the most essential proteins encoded for the RSV viral genome. The G protein is the most varying structure among RSV strains, and this variability dictates the antigenic nature between RSVA and RSVB [9]. The G protein is a 298 amino acid glycoprotein that is involved in the attachment of the virus to the host cell [5,13]. The G protein targets the ciliated human airway epithelial cells for virion attachment, specifically the nasal epithelium [3,8]. The G protein exists in both a membrane-bound form (mG) and a secreted form (sG) [8]. In a medium of RSV-infected cells, approximately 80% of all the total released G protein is present as sG. In comparison, the remaining 20% is present as mG incorporated into the virion particles [21]. The membrane form (mG) is anchored by a transmembrane domain near the N terminus [8,21]. The sG form, however, is important in capturing antibodies generated by the host against this protein and, thus, preventing the opsonization and neutralization of the virus by G-specific antibodies [13,21]. sG has been shown to interfere with antibody-mediated neutralization, acting as an antigen decoy and impeding cell-mediated neutralization of RSV by Fc receptor-bearing immune cells [21]. The ability of sG to evade the host-generated antibodies against itself is how the G protein evades the host’s immune system and increases the host’s infectivity. Another vital feature of the G protein is its capacity to impair the function of chemokine and cytokines due to a CX3C chemokine-like motif that can imitate and compete with these molecules for the interaction with their receptors, which can also modulate CD8+ T cell responses [13]. The CX3C motif noted has been reported to reduce the influx of immune cells into the lungs of RSV-infected mice [5]. Moreover, it has been shown that the G protein shares structural similarities with the tumor necrosis factor (TNF) receptors. As a result, it is likely to interact with TNF family cytokine members, conducting an imbalance in the inflammatory response that is mediated by these molecules [8,13]. These findings indicate that the G protein has numerous roles. Apart from the role of viral attachment to the host cell, G proteins play a crucial role in modulating the immune response triggered by the RSV infection. Modulation of the immune system triggered by the RSV ultimately renders the immune system incompetent against the infection.

The F protein is the second of the two viral neutralization antigens of the RSV genome. The F protein is highly conversed between RSVA and RSVB, with less than 10% sequence diversity between the two groups [13]. The 574 amino acid F protein directs viral penetration and syncytium formation, like a typical F protein of Paramyxoviridae [5]. To be competent enough to cause fusion of the viral particles and the host cells, the F protein must be cleaved at two polybasic sites separated by 27 amino acids. This proteolysis generates two subunits, the carboxy-terminal F1 and the amino-terminal F2 subunit [13]. A heterodimeric promoter is created when the F1 and F2 subunits are covalently connected by two disulfide links. Three of these promoters combine to create the mature trimeric version of the F protein [9,16]. The viral particle's active F protein is a trimer in a perfusion conformation [13]. After engaging with the receptor, the F protein will change its shape to a post-fusion form in this trimeric state, which will mediate the fusing of the viral envelope with the host membrane [8,13]. According to recent research, one of the most significant RSV proteins involved in infection and host cell contact is considered to be the F protein.

The SH protein is a surface membrane on the RSV virion and has been shown to display two different forms in varying sizes depending on whether it is in RSVA or RSVB [13,22]. RSVA is 64 amino acids in length, and RSVB is 65 amino acids [22-23]. The SH protein has been described as a viroporin, capable of forming ion channels in cellular membranes [22]. The SH protein is not involved in viral entry into host cells; however, it may work as a virulence factor during the RSV infection [13, 22-23].

Whether the protein plays a role in the virion packaging, attachment, and entry into the host cell or evades the host’s immune system, each viral proteins play an integral role in the infectious nature of RSV. Many proteins, both structural and non-structural, are imperative in ensuring the host’s immune system is impaired or evaded completely to ensure that the virion packaging occurs appropriately. Though studies show some of these proteins are not necessary for the infectious nature of RSV, each viral protein plays a pivotal role in ensuring the overall pathophysiology of the virus. 

History and epidemiology

As reported in numerous studies, RSV was first discovered in a colony of chimpanzees with throat swabs in 1956, exhibiting symptoms like sneezing, coughing, and purulent nasal discharge [24-25]. These similar symptoms were also quickly observed in the other monkeys within the colony, suggesting that the pathogen in question was highly contagious [24]. Due to its origin, the viral pathogen was initially called Chimp Coryza Agent [25]. However, later in 1957, a similar viral agent was isolated from the throats of infants with pneumonia and croup and was renamed RSV [24-25]. More than six decades later, RSV remains the most common viral cause of serious severe respiratory illness in children under five years of age and the major cause of infantile bronchiolitis [26]. Later on, the bimodal pattern of RSV was discovered as cases amongst the elderly were reported. As mentioned previously, the infection caused by RSV is a seasonal disease for which the onset, peak, duration, and severity vary somewhat from country to country and year to year [27]. In temperate climates, RSV activity typically peaks during the winter months, although the actual peak of the season may vary by region [26-27]. In tropical regions, RSV tends to peak during the rainy season in the hottest months [27]. These seasonal patterns before 2020 were very consistent. However, the circulation patterns of RSV and other common respiratory viruses have been disrupted since the start of the COVID-19 pandemic in early 2020 [2]. Beginning in the southern region of the United States, RSV circulation began to rise in the spring months of 2021 and peaked in July 2021 [2,28]. Due to the widespread distribution of RSV, at least 50% of infants in the United States have contracted RSV and almost all children have contracted it by the time they turn two years of age [29].

When focusing on the transmission of RSV, the concept of R_0__ _becomes very important. R_0_ is referred to as the reproductive ratio, or in other words, the degree of transmissibility of the virus. The R_0_ represents how many people can be infected by one person [30]. The R_0_ for RSV varies anywhere from one to five; however, it is typically estimated to be three [27-28]. The variability of R_0_ is primarily due to several factors including, but not limited to, environmental, biological, and behavioral factors. The R_0_ of RSV may vary annually and seasonally, which may also be attributed to the antigenicity of the strain circulating in that particular year. While it may not be solidified, RSVA has been denoted to have higher transmissibility, and therefore, the R_0_ for RSVA may be higher than that of RSVB. Knowing the R_0__ _of RSV is important to ensure proper techniques to prevent virus transmission. Like many other respiratory viruses, the steps to prevent the spread of RSV are fundamental. Avoiding close contact, frequent sanitation of contaminated surfaces, and washing hands with soap and water for at least 20 seconds can decrease the transmission of RSV. Covering the mouth while sneezing and coughing can also limit the spread of the virus. Despite the overall preventative measures, RSV causes a minimum of 3.4 million hospitalizations in the United States annually [2,13]. The burden of RSV infections is increasing due to rising hospitalization rates and higher healthcare costs [13].

## Review

The clinical presentation of RSV largely depends on the age cohort infected, which is imperative to understand as healthcare providers. In infants and children, RSV is most likely to present as viral bronchiolitis. In adults, RSV typically manifests as upper respiratory tract infections with mild to moderate symptoms, only rarely causing severe disease [31]. Differences in host immunity, anatomy, and other risk factors lead to different symptomatic profiles occurrence.

Risk factors

The list of risk factors affecting RSV pathogenesis varies greatly. Furthermore, the presentation of multiple risk factors increases the likelihood of developing a severe RSV disease. The risk factors also increase the likelihood of RSV-induced complications. Risk factors can be categorized as host risk factors and environmental risk factors, which equally contribute to RSV illness. Several host factors that lead to hospitalization are preterm birth, malnutrition, gender, chronic lung disease (CLD) also known as bronchopulmonary dysplasia (BPD) of prematurity, frail old age, congenital or acquired immunodeficiencies, trisomy 21, and inborn errors of metabolism, amongst many other conditions that increase the vulnerability of an individual with an RSV infection [24,32]. Each of these factors alters and impairs the host’s immune system, causing an increased susceptibility to develop other conditions. RSV is also more likely to occur in infants and children with cystic fibrosis, recurrent aspiration pneumonia, tracheoesophageal fistulas, and neurological or genetic conditions that prevent good secretion and respiratory clearance [29]. Preterm birth and other underlying medical conditions not only increase the risk of hospitalization, but also indicate a more frequent requirement for mechanical ventilation, admission to the intensive care unit (ICU), longer duration of hospitalization, and increased mortality [32].

Environmental factors such as tobacco exposure can also increase RSV susceptibility. Despite the vast list of risk factors, the most frequently and consistently identified risk factors include young age (<6 weeks), male sex, siblings or other children living in the household, mainly when they are older and already attending daycare or school, the infant’s daycare attendance, and exposure to passive smoke in the home, and crowded living conditions [24,29,31-32]. RSV can also be contracted as a nosocomial infection, primarily due to the infectious nature of the infection.

Age continuously remains the most significant risk factor. Children have smaller diameter airways, impaired respiratory capacity, and a lower respiratory reserve [31]. Thus, obstruction of the small airways in infants has a greater clinical significance than an obstruction in the peripheral airways of an older child or adult [33]. Whether premature or not, RSV illness is likely to be more severe in male infants which is largely due to the smaller diameter of their airways compared to female infants. Preterm birth is another significant risk factor for RSV illness, largely due to the development of a premature immune system. Naturally, the body responds to an RSV infection by mounting an immune response that produces RSV-specific antibodies of the IgG, IgM, and IgA types, which then can be found in both serum and airway secretions [29]. Preterm infants are more likely to contract RSV infection due to the lack of passive immunity provided by the mother's antibodies, which are crucial to protect against infections in the first few months of life [24].

Malnutrition was also noted as a host risk factor, specifically vitamin D deficiency. Vitamin D plays a major role in innate immunity and influences the lung function of asthmatic patients [24,31]. In its active form, vitamin D 25-hydroxyvitamin [25(OH)D] helps to modulate inflammatory processes. It has been reported that children and adults with low concentrations of 25(OH)D have an increased risk of contracting a severe respiratory infection or exacerbating asthma [24]. Inamo and colleagues indicate that the risk of contracting a severe respiratory infection in children and infants rises when 25(OH)D concentrations fall below 75 nmol/L(30 ng/mL), making these children more vulnerable to bacterial and viral lung infections [24,34-35]. It has been reported that low levels of vitamin D in the cord blood of healthy newborns are associated with a greater risk of severe RSV lower respiratory tract infections during the first year of life, suggesting that the mother's low vitamin D intake during pregnancy may also affect infants' severity of the disease [24].

While most research is focused on the risk factors associated with RSV and their effects on infants, many of the same risk factors can be attributed to increasing vulnerability to illness in the elderly. In the elderly cohort, the most significant risk factors are pulmonary disease, especially chronic obstructive pulmonary disease (COPD), and functional disability as measured by activities of daily living [31]. Interestingly, while coronary artery disease and diabetes are risk factors for severe influenza, they are not associated with an increased risk of severe RSV disease [31,34]. The care home residents are more likely to develop RSV, as 12% of all RSV admissions occur in care homes, and the mortality rate is 38% in this group compared to 3% in patients admitted from the community [31]. Declining immune systems and lower RSV-specific serum immunoglobulins and nasal IgA titers are associated with susceptibility to RSV in the elderly [6]. The elderly may be more vulnerable because of a blunted immune response, as is in transplant recipients, although through different mechanisms [6,34].

Immunocompromised children also have a much greater likelihood of a severe RSV infection. Infants exposed to high levels of particulate air pollution and chronically exposed to cigarette smoke are also more likely to suffer more severe RSV infections [24,29]. As mentioned previously, hospital staff appear to be a major source of nosocomial RSV infections, and among this staff cohort, these infections usually manifest as a cold or flu-like illness [29]. Individuals that are considered to be at the highest risk of RSV infections are medical students and staff that are new to the unit area. Due to this, all staff and medical students must follow proper prevention techniques to ensure transmissibility is as low as possible.

Transmission and life cycle

As with many other respiratory viruses, RSV transmission occurs via large droplet inoculation in the eyes, nose, or mouth, requiring close contact with an RSV-infected subject or auto-inoculation to the face (nose, mouth, or eyes) via contaminated fomites or skin [36]. Transmission of large droplets may occur from the nose and mouth of an infected individual as they cough or sneeze. As an estimate, a single cough is estimated to produce up to 3,000 droplets [30]. Not only can these droplets directly infect another individual, but they can also land on several types of surfaces. Depending on the surface type, the virus may be active and infectious for various periods.

**Figure 3:**
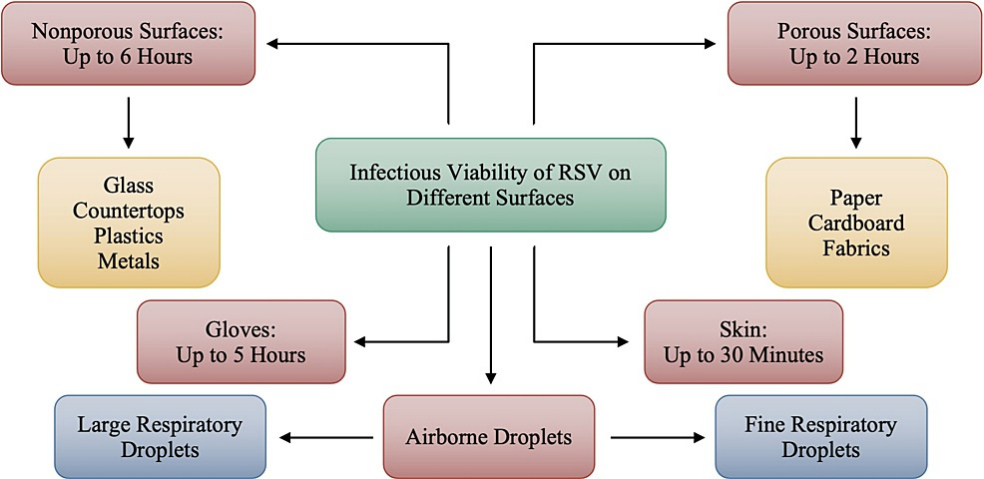
Infectious Viability of Respiratory Syncytial Virus (RSV) on Different Surfaces Image credits: Jasndeep Kaler, Azhar Hussain, Kishan Patel, Tamara Hernandez, Sidhartha Ray

Research suggests the viability of RSV is the longest on nonporous surfaces for up to six hours. As noted in Figure 3, nonporous surfaces include hard surfaces such as countertops, glass, plastics, and metals. Varnished wood is also considered a nonporous surface; however, the viability of RSV on varnished wood has yet to be studied. On porous surfaces such as paper, cardboard, and fabrics, the viability of RSV is as long as two hours. On gloves, the virus is active for up to five hours and due to this timeframe, it can be understood why RSV can be transmitted through hospital wards and other healthcare facilities as a nosocomial infection. RSV is active for the least amount of time on the skin, for approximately only 30 minutes. As with all respiratory viruses, RSV can also be transmitted through airborne droplets. However, as depicted in Figure 3, the duration of these airborne droplets’ viability is unknown.

Respiratory viruses are thought to be transmitted via multiple modes of transmission, sometimes divided into three categories: contact, large respiratory droplets, and fine respiratory droplets, with the latter sometimes also referred to as aerosol or airborne transmission [37]. The size of the respiratory droplets is also important, as the fine respiratory droplets can be generated during coughing, sneezing, talking, or exhaling [37-38]. In comparison, large droplets settle quickly, whereas smaller aerosols can remain airborne and may transport over longer distances by airflow [37]. Smaller aerosols are also more likely to be inhaled deep into the lung and cause infection in the alveolar tissues of the lower respiratory tract. In contrast, larger droplets are more likely to be trapped in the upper airways [37,39-40]. While the size of aerosol droplets has not been thoroughly investigated because the smaller aerosol droplets can travel deeper into the respiratory tract, it can be assumed that more severe RSV disease can occur from fine airborne droplets in comparison to the indirect transmission through intermediate surfaces. The exact particle size threshold used to differentiate between droplets and aerosols has yet to be agreed upon. However, according to the World Health Organization (WHO) and Centers for Disease Control and Prevention (CDC), the disease transmits through droplet transmission which is particles greater than 5 mm in size, and aerosol transmission in which the particle size is 5mm or less [37,38]

When RSV is inhaled through mucous membranes such as the nose or mouth, it infects airway epithelial cells (AECs) in the upper respiratory system. From there, it spreads to the lower respiratory system and reaches the bronchioles, where viral replication is more effective [24]. The site of the viral attachment to the host cell is generally in the nasal epithelium [29]. Ciliated cells in the bronchial epithelia and type I pneumocytes in the alveolus are the primary cells targeted by RSV infection [4]. AECs with polarized, ciliated edges begin to become infected by RSV after the virus is attached to their apical surface [9]. As discussed before, the G protein is associated with the cell surface factors and facilitates the initial attachment step [9,13]. The G protein interacts with heparan sulfates and chondroitin sulfate B glycosaminoglycans (GAGs) [13]. After this interaction, which helps the virus approach the cell membrane to be infected, the F protein contacts its receptor, nucleolin [9,13]. Once fusion has occurred, the helical ribonucleoprotein complex (RNP) is released into the host cell cytoplasm [2,9]. The transcription of viral mRNA and the synthesis of positive-sense anti-genome intermediates are required for the replication of new negative-sense genomes to pack into visions that are carried out by the viral RNA-dependent RNA polymerase (RdRp) complex [9,41].

Once the entire replication process has been completed, RSV virions will be assembled at or near the plasma membrane. Initial models have proposed that F proteins bind to lipid rafts, recruit and concentrate M proteins via the F cytoplasmic trail. By actin-dependent membrane deformation directed outward, this process starts filament budding [41]. However, other studies have suggested that this procedure may be more intricate, with filament formational starting intracellularly as vesicles containing viral proteins move along microtubules and develop into filaments that are later loaded with nucleocapsids before fusing with the plasma membrane by an unidentified mechanism [9,41-42]. After budding from the apical membrane of polarized epithelial cells, virions detach followed by their release in an M-dependent maturation process as a filamentous particle approximately 130nm in diameter and 0.5-1.2 micrometers in length [42]. Once virion particle replication has occurred, the host is considered infectious and possesses the ability to infect other individuals. Infected individuals will remain contagious as long as the virus is being shed. Shedding of the virus begins within a day or so of infection, often before the onset of major symptoms [29]. Viral shedding is highly variable and appears to correlate roughly with the age of the infected host, the severity of the infection, and whether or not the infected individual is immunocompromised [5,29]. Typically, adults will shed the virus for 3-7 days following the infection [29]. Infants generally shed for up to 14 days in mild infections, however, infants less than six months of age with severe infection may shed for three weeks [29,31]. In contrast, immunocompromised individuals may shed for several months following infection [29].

Once an RSV infection has begun, the respiratory tract's epithelial cells undergo severe deterioration. If the damage is restricted to upper airway cells, the symptoms resemble those of an upper respiratory infection [29-30]. The infection frequently spreads to the lower respiratory airways in previously uninfected individuals, typically infants, and immunocompromised individuals, resulting in the classic symptoms of a lower respiratory tract infection [1,29]. As a result, the capillaries become more permeable, there is an increase in the secretion, and chemokine draws more pro-inflammatory cells such as histamine, interleukin 1 and 6, macrophages, neutrophils, eosinophils, and natural killer (NK) cells to the site of infection [29]. Increased capillary permeability in interstitial areas, small areas, and alveoli result in a leakage of plasma proteins. The leakage of the plasma proteins into these areas will lead to generalized interstitial swelling and is also noted to inhibit pulmonary surfactant function [29,43]. Pulmonary surfactant reduces surface tension, thereby preventing the structures from collapsing at the end of expiration. Surfactant also reduces the work that is associated with respiration.

Furthermore, the main functions of surfactants also include interacting with and subsequently killing pathogens or preventing their dissemination and modulating immune responses [44]. In illnesses such as RSV, where the function of pulmonary surfactant has been inhibited, an infected individual will find themselves in respiratory distress. Moreover, leukotrienes C4 and D4, two pro-inflammatory mediators that are strong bronchoconstrictor, have also been identified from secretions of people with severe lower respiratory tract infections [29,43]. Due to a dysfunctional mucociliary elevator, increased secretion production, poor secretion clearance, inadequate surfactant activity, and small airways getting clogged due to secretions and cell debris [29]. Small airways can become narrow ever further as a result of bronchoconstrictor substances, increasing airway resistance, air trapping, and wheezing, all of which are signs of severe RSV infections of the lower respiratory tract [29,43]. The presence of potent bronchoconstrictor and pro-inflammatory mediators lead to the clinical symptoms noted in RSV-infected patients. The pathogenesis of the disease is very well described in young children; however, little is known about the pathogenesis for older adults. Similar pathogenesis may occur with subtle differences due to immune status and immunosenescence. Reinfection is also very common due to limited immunity. Reinfection can occur annually; however, it can also occur much more frequently, even within a few weeks of the previous infection.

**Figure 4:**
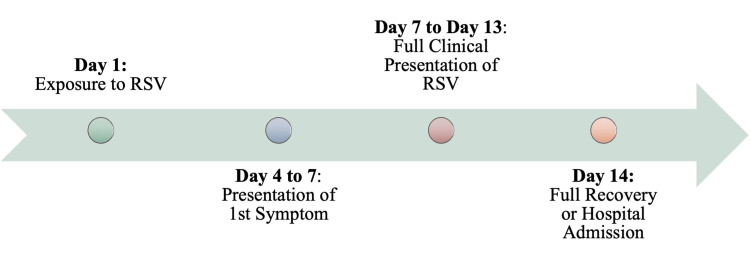
Median Timeline from Respiratory Syncytial Virus (RSV) Infection to Presentation of the First Symptom Image credits: Jasndeep Kaler, Azhar Hussain, Kishan Patel, Tamara Hernandez, Sidhartha Ray

Though the presentation of symptoms varies from individual to individual, depending on the cohort infected, the median timeline of RSV remains steady. Figure 4 provides a schematic representation of the timeframe from exposure to RSV to the presentation of RSV-associated symptoms. Individuals will typically develop RSV symptoms four to seven days after being exposed to RSV. This incubation period may also be referred to as the eclipse phase [4]. An individual in the eclipse phase may not present with any symptoms; however, these individuals are still contagious for one to two days before the symptomatic presentation occurs [4,45]. It is crucial to keep in mind that while up to 40% of people with RSV will be asymptomatic, they may still be contagious. Understanding the eclipse phase is important since during this time that an infected individual is most likely to transmit the viral infection to others. As the first symptoms arise, the virus has already begun rapidly disappearing from the system [4]. As with other viral respiratory illnesses, RSV is self-limited, and most individuals will begin to feel better after about two weeks.

In some cases, the occasional cough may persist for more than a few days to a few weeks. In high-risk individuals, instead of full recovery, an individual may be hospitalized due to illness becoming more severe. In some cases, it is by this time that complications may also arise in premature infants, infants, the elderly, or the immunocompromised. While most individuals are usually contagious for three to eight days, some infants, and people with weakened immune systems can continue to spread the virus even after the symptomatic presentation resolves for as long as four weeks [2]. 

Clinical manifestation and RSV-associated complications in infants

**Figure 5:**
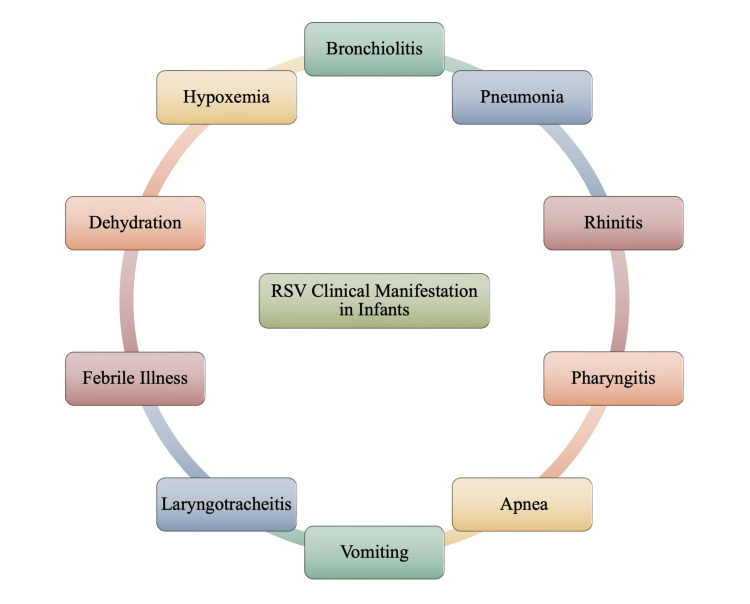
Clinical Manifestations of Respiratory Syncytial Virus (RSV) in Infants Image credits: Jasndeep Kaler, Azhar Hussain, Kishan Patel, Tamara Hernandez, Sidhartha Ray

The initial symptoms of RSV are similar in all groups, but the course of the illness differs. Patients will typically experience mild to moderate nasal congestion and low-grade fever within a few days of exposure and transmission, followed by a productive cough within a few days [29]. Patients with previous RSV infections may experience these symptoms for several weeks and then resolve without further incident [1,29]. All individuals will have symptoms mimicking an upper respiratory tract infection; however, the symptomatic profile afterward may differ. Lower respiratory tract infections are more frequently seen in infants 2-3 days after the onset of upper respiratory tract infection signs and symptoms [29]. Infants and children with this infection can present with a variety of symptoms, ranging from mild upper respiratory tract symptoms to life-threatening lower airway involvement that requires hospitalization and mechanical ventilation [1]. Figure 5 provides a visual of the varying clinical symptoms that an infected infant may have present.

Viral bronchiolitis is one of the most common infant viral illnesses that occur as a result of RSV infection. In approximately one-third of infants with viral respiratory infections, progression to lower respiratory tract involvement is observed. While bronchiolitis is the most common progression, pneumonia and laryngotracheitis (croup) may also develop [1,29]. Bronchiolitis typically develops after an initial prodrome of nasal congestion, cough, and coryza lasting up to three days [31]. Subsequently, a low-grade fever, wheezing, crepitations on auscultation, and signs of increased respiratory effort develop, such as nasal flaring, chest wall retraction, and tachypnea [1,31]. RSV-associated apnea is also very prominently noted in infants under six months of age and is a vital indicator for hospital admission [31]. In some infants, coughing may become more severe and as such, secretions are noted as being more copious and thicker, leading to the presentation of pharyngitis. Pharyngitis is noted amongst at least one-quarter of the infants. On examination, rhinitis, and pharyngitis may be associated with conjunctival signs and erythema to the tympanic membrane [1]. Vomiting is also commonly noted in about half of the affected individuals [1,29,31]. Very young infants may present only with lethargy and poor feeding, which may lead to hospitalization due to dehydration and potential malnutrition. Such infants usually present with severe hypoxemia and dehydration that may have aspiration prior to their visit to a physician’s office or hospital emergency room [29].

**Figure 6:**
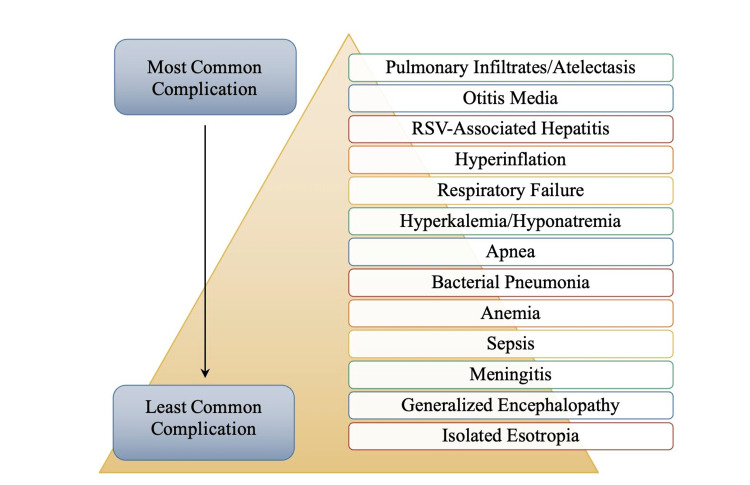
Most to Least Common Complications of Severe Respiratory Syncytial Virus (RSV) Infection in Infants Image credits: Jasndeep Kaler, Azhar Hussain, Kishan Patel, Tamara Hernandez, Sidhartha Ray

Furthermore, a chest radiograph typically shows hyperinflation with a flattened diaphragm; a radiograph may not be the primary choice of diagnosis in bronchiolitis. While extrapulmonary manifestations of RSV are uncommon, Jha et al. noted that seizures, hyponatremia, cardiac arrhythmia, cardiac failure, and hepatitis have been noted in infants with RSV infection [1]. Long-term complications associated with RSV have been noted in infants and young children. Alongside the significant burden of acute RSV infection, there has been an association between RSV infection as an infant and subsequent pulmonological symptoms that sometimes persist into adulthood [1,31]. Figure 6 illustrates various complications of RSV that have been observed in infants, ranging from most common to least common.

Pulmonary infiltration and atelectasis are the most common complication of severe RSV infection in infants. Consolidation with pneumonia can be due to the virus or a secondary bacterial infection. Furthermore, an association between a severe infantry RSV infection and subsequent wheezing, allergy, and asthma, persisting into adulthood, has been noted [1]. Another common complication of RSV in infants is otitis media, which can result from erythema to the tympanic membrane and, less frequently, bulging of the tympanic membrane. As mentioned earlier, hyperinflation of the lungs may be noted on a chest radiograph because of bronchiolitis, which may lead to respiratory distress that persists later in life. Acute neurological signs and symptoms such as central apneas, seizures, lethargy, feeding or swallowing difficulties, abnormalities of tone and strabism, and abnormalities of the cerebrospinal fluid (CSF) were noted in 39% (n = 121) of RSV-positive patients on the pediatric intensive care unit (PICU) [46-47]. The olfactory receptor gene OR13C5 has also been referred to in RSV pathogenicity, though the exact mechanism of involvement is not fully understood. The olfactory nerve connects to the nasal cavity with the central nervous system (CNS) and thus could be used as a shortcut for RSV [24]. This presumed shortcut could explain the neurological symptoms and complications that are produced by RSV.

Though these symptoms and complications occur in at least 2% of RSV-infected individuals, most severe, and permanent neurological conditions may occur. Strabism has also been reported as a neurological complication in the form of esotropia in four of 12 patients in a study conducted by Eisenhut [47]. Esotropia is a type of strabismus in which one or both eyes turn inward. It is unknown whether esotropia will resolve independently or is more permanent. Hyponatremia is diagnosed when an individual’s serum sodium level of less than 136 mEq/L is observed. Hyponatremia is a complication found in 33% of infants requiring intensive care with RSV infection, as noted by Hanna and colleagues [46-48]. Hyponatremia (sodium < 115 mEq/L) in infants may also lead to the presentation of seizures which may be the cause of why some infants may present with generalized tonic-clonic seizures and why others may present with partial seizures with altered consciousness and focal motor features or eye deviation [47-48]. Elevated transaminase levels have been found in 46% to 49% of ventilated children with RSV bronchiolitis [47]. RSV-associated hepatitis is a complication that is more prevalent in children with congenital heart disease [24].

Clinical manifestation and RSV-associated complications in the elderly and immunocompromised

**Figure 7:**
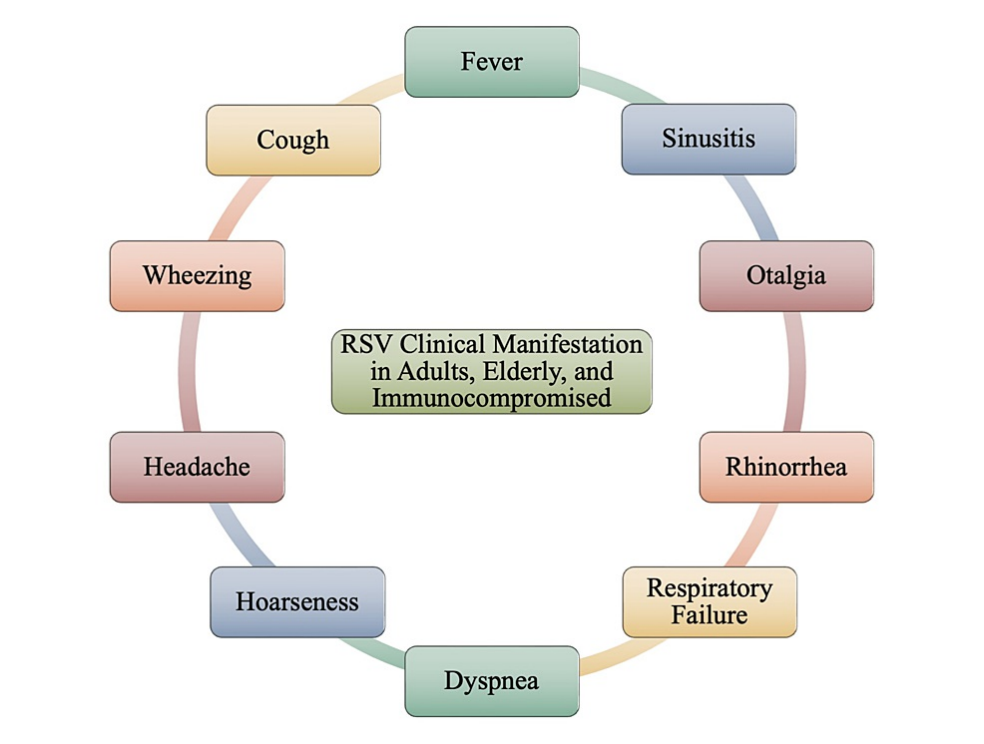
Clinical Manifestations of Respiratory Syncytial Virus (RSV) in Adults, Elderly, and Immunocompromised Image credits: Jasndeep Kaler, Azhar Hussain, Kishan Patel, Tamara Hernandez, Sidhartha Ray

In elderly patients, the symptomatic profile is similar to that seen in infants, although with greater severity and increased likelihood of lower respiratory tract involvement. This can be attributed to a much more delayed diagnosis of RSV in adults, as individuals may associate the early non-specific symptoms with influenza or the common cold. Typically, an RSV illness in adults will begin with nasal congestion before progressing to a cough [1]. Audible crackles will also be noted on a chest examination, and infiltrates will be noticeable on chest radiographs in up to half of the elderly patients [1,35]. Many of the RSV-associated symptoms in older adults and the elderly include non-specific features of a viral respiratory tract infection such as coryza, sore throat, fever, and malaise, as well as lower respiratory tract symptoms such as cough, as noted in Figure 7 [1].

Furthermore, RSV pathogenesis in adults and the elderly are also different from that of infants. A study of adults between the ages of 24 and 95 years exposed to RSV, showed a detectable virus for 10 to 13 days in nasal secretions that in some cases lasted for at least 20 days [34]. In adults, it is required that a diagnosis of RSV infection based on laboratory diagnostic tests be confirmed due to the similarity of RSV symptoms with other viral and bacterial agents that cause acute respiratory tract infection [34,36]. Initially, RSV was identified as a common viral infection of the respiratory tract. RSV was not recognized as a potentially serious problem in older adults until the 1970s when outbreaks of the virus occurred in long-term care facilities. Since then, additional studies in hospitalized adults have suggested that RSV may be an important cause of illness in community-dwelling elderly people [6]. Clinical presentation observed in adults and the elderly can vary from cold-like symptoms to acute respiratory distress [36]. After 3 to 4 days, lower respiratory tract symptoms such as cough, sputum, wheezing, and dyspnea are observed in a significant proportion of RSV-positive adults [34,36]. Wheezing, like in infants, may also be noted in adults. In cases where RSV is similar to a common cold with nonspecific symptoms, it is unlikely to cause a significant fever, giving RSV-positive patients no significant signal to self-isolate. As such, these individuals may continue about day-to-day life without knowing that they are infectious and, thus, may unknowingly spread RSV to others who may be more vulnerable to much more severe outcomes.

**Figure 8:**
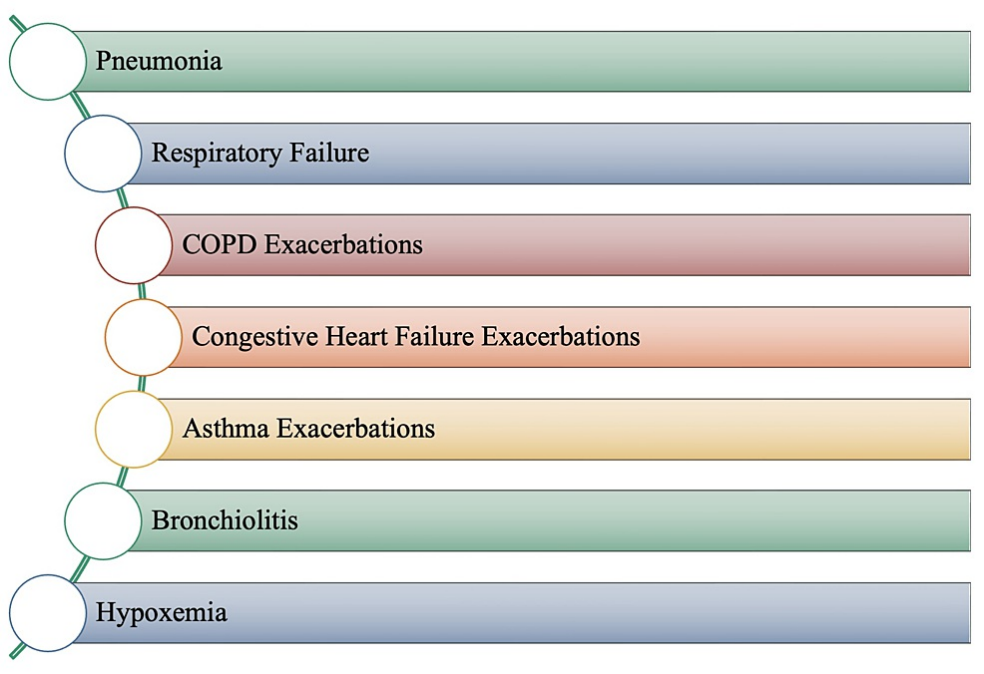
Respiratory Syncytial Virus (RSV) Associated Complications in Adults, Elderly and Immunocompromised Image credits: Jasndeep Kaler, Azhar Hussain, Kishan Patel, Tamara Hernandez, Sidhartha Ray

Immunocompromised RSV-positive patients are predisposed to otalgia. Therefore, clinical or radiological evidence of sinusitis in immunocompromised individuals can help distinguish RSV from other viral causes of infection, such as cytomegalovirus (CMV) [1]. The clinical progression of RSV infection in immunocompromised adults appears to follow a similar pattern as in immunocompetent hosts, with upper respiratory infection preceding lower respiratory tract disease [6]. While RSV infection in the immunocompromised adult is associated with significant morbidity and mortality, the severity of clinical manifestations depends on the magnitude of the immunosuppression, with bone marrow transplant recipients at the greatest risk of complications [1,6,31]. As mentioned previously, while little is known about the presentation and complications associated with RSV infection in older adults and the elderly, Figure 8 depicts the most noted complications.

There is a gradual deterioration of the immune system due to aging, referred to as immunosenescence. In addition to having decreased immune function, elderly people have weaker respiratory muscles and diaphragms, further limiting lung expansion [34]. Additionally, older adults have decreased levels of protective mucus, lung compliance, and elastin [6,34]. This is one of several reasons why older adults are at an increased risk of complications following a viral infection, such as RSV.

Pneumonia and bronchiolitis are more prevalent among infants and younger children but may also be noted in older adults as severe forms of lung infection. RSV infection can also cause exacerbations of COPD, congestive heart failure, and asthma. Furthermore, lung damage caused by COPD is not retrogressive; respiratory function will further decrease with an RSV infection. In some cases, the COPD exacerbations caused by RSV may be severe enough to warrant hospitalization and require monitoring. A similar mechanism can be proposed for congestive heart failure exacerbations. Because an individual diagnosed with congestive heart failure will already present with compromised respiratory function, RSV infection will exaggerate the patient’s symptomatology and, thus, may even result in mortality. Individuals with asthma will also present with exacerbated symptoms when infected with RSV. An asthmatic individual who is also RSV positive may probably encounter asthma attacks more frequently. In addition, with severity, the permanent narrowing of the airways would trigger difficulties with respiration that may not deter upon recovery from the infection. In some cases, these exacerbations may be so extreme that respiratory failure and death may result from an RSV infection.

Clinical management and treatment

**Figure 9:**
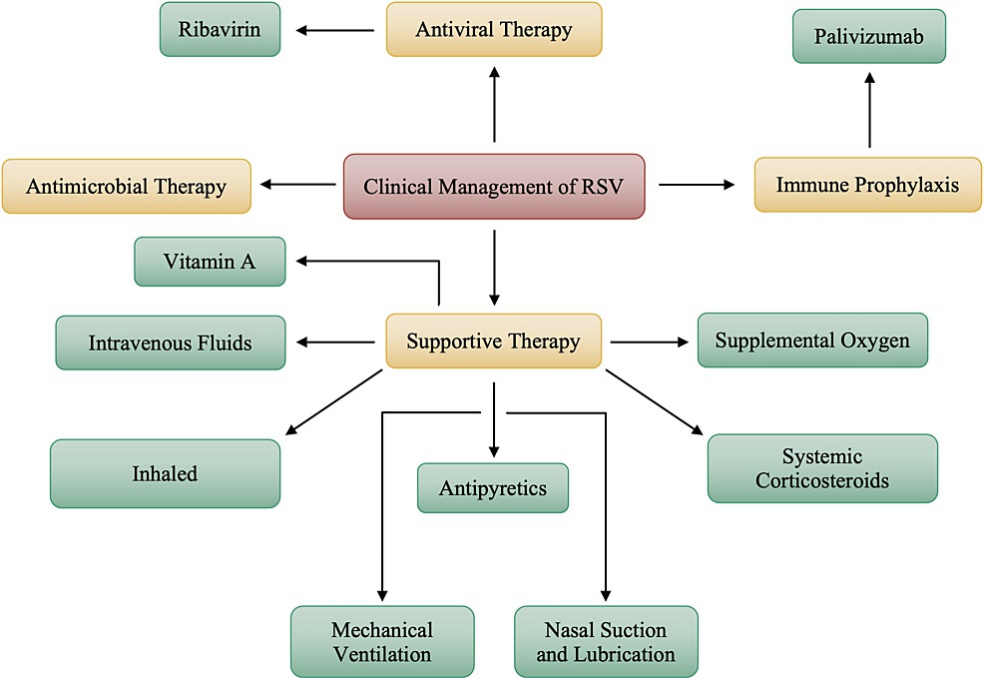
Clinical Management and Treatment Options for Respiratory Syncytial Virus (RSV) Image credits: Jasndeep Kaler, Azhar Hussain, Kishan Patel, Tamara Hernandez, Sidhartha Ray

Due to the record-breaking nature of RSV cases in recent years, there is a greater emphasis on the supportive nature and the symptomatic management, all while attempting to prevent transmission. In most cases, the duration and symptomatic presentation of RSV is self-limited and symptomatic management is the course of action. However, the standard care of clinical management of RSV infection differs depending on whether the infection is in adults or children. Most infants and children infected with RSV are successfully treated as outpatients [31]. Hospitalization is much more likely in the elderly and the immunocompromised due to the presence of any pre-existing conditions that may increase the severity of the RSV illness. Premature infants may also require hospitalization. Presently, options for antiviral treatment are very limited; however, antiviral therapy is considered and used more frequently in lung transplants, immunocompromised status, and hematopoietic stem cell transplantation (HSCT) [3]. Antiviral drugs, such as aerosolized ribavirin, are recommended in severe pediatric presentations [36]. As with all viral illnesses, the clinical management of RSV consists mostly of supportive therapy, as noted in Figure 9. However, antiviral intervention may occur in severe cases.

In cases of superimposed bacterial infections, antibiotics may also be administered; however, they are not considered the first line in treating and managing RSV. In patients with RSV bronchiolitis, the chance of developing a subsequent bacterial infection is extremely low; thus, routine use of antibiotics in treating RSV bronchiolitis is not warranted [45]. The decision as to whether antibiotics must be administered or not depends on the patient’s clinical presentation, which may suggest the presence of bacterial superinfection. The typical laboratory findings that focus on determining whether antibiotics should be administered are the white blood cell count (WBC) and c-reactive protein (CRP). A WBC count greater than 15x109/l has been associated with bacteremia [4,45]. While WBCs may increase up to 20x109/l, serum CRP levels in RSV infections are usually low [45]. Due to this, the use of antibiotics tends to be restricted to children in whom an unexpected clinical deterioration is accompanied by an increase in inflammatory parameters such as CRP [4,45].

Pharmaceutical approaches to develop an effective cure for RSV infections have been ongoing for as long as the virus has been recognized; however, no effective treatment beyond supportive therapy has appeared [29]. Supplemental oxygen is administered in individuals exhibiting the typical signs of lower respiratory tract infection [1,29]. Supplemental oxygen acts to maintain a saturation of 92%; however, it is only considered in severely ill hospitalized patients [29]. Alongside oxygen administration, intravenous (IV) fluids may also be required in hospitalized infants in which dehydration or poor feeding is indicated [46-49]. To prevent aspiration, oral feeding should be withheld in patients with high respiratory rates or distress [4]. Mechanical ventilation may be considered in those with either respiratory failure, severe apnea, or both [46]. Antipyretics may be administered to decrease the fever in RSV-positive of all ages. Unlike in adults, respiration in infants is typically through the nasal cavity. Nasal congestion and rhinorrhea are part of the clinical manifestation amongst infants, and due to nasal congestion, breathing in infants becomes impaired and difficult. Nasal suction and lubrication are used to relieve nasal congestion and may also be utilized, predominantly in infants, to aid respiration [45].

While the effects of inhaled bronchodilators and systemic corticosteroids as supportive therapy in RSV remain controversial, they are frequently prescribed in patients with underlying lung diseases such as asthma or COPD presenting with acute exacerbation associated with bronchospasm and wheezing [34]. While there are no reports indicating differences in viral shedding or cell-mediated immunity, humoral immunity is likely diminished with the use of systemic corticosteroids. Therefore, the potential benefits should be weighed against the severity of the symptoms [29,34,36]. Bronchodilators may be used more frequently in children with RSV infections. The hallmark of airway obstruction in RSV-induced bronchiolitis have been associated with necrosis and lysis of respiratory tract cells, edema of the submucosa, increased mucus secretion with or without bronchial hyperreactivity, and surfactant abnormalities associated with airway collapse [35]. β-agonists generate bronchodilation and contribute to mucus clearance and the production and release of surfactants [29,35]. Mucus clearance and the production and release of surfactant can decrease the workload on the lungs that is associated with respiration and, thus, decrease the likelihood of respiratory distress or respiratory failure. Several studies failed to demonstrate a significant improvement in oxygen saturation levels, and in some cases, β-agonists were reported as inducing bronchospasms [35].

In some cases, vitamin A may be administered as a supportive therapy, though it may not decrease respiratory morbidity in children with acute RSV bronchiolitis. According to research, children with RSV infections had considerably lower serum vitamin A levels, which might have contributed to an infection that progressed more severely [29,45].

While several different drugs have been shown to reduce RSV infection, currently, only ribavirin is allowed for human use. However, ribavirin is no longer included in the American Academy of Pediatrics (AAP) guideline for the management of RSV bronchiolitis in children [3,29,35,45]. Ribavirin is a guanosine analog that is FDA-approved in its aerosolized formulation [3]. After administration, ribavirin is phosphorylated to ribavirin triphosphate, the highly active metabolite [45]. This active metabolite is then catabolized to triazole carboxamide, which is the inactive metabolite that is excreted through urine [45,49]. Ribavirin is most commonly administered through a nebulizer. Multiple studies suggest that early treatment with ribavirin is much more effective than later during the clinical presentation.

Ribavirin is a virostatic agent that inhibits viral replication. As mentioned earlier, by the time the first symptoms of RSV emerge, the virus is already rapidly disappearing from the system. Due to the nature of the virus, the effect of ribavirin depends on how long after the initial infection the drug is administered. The first symptoms of RSV are observable after five to seven days. If ribavirin is administered at this point, the pediatrician has provided a virostatic agent in cases where the virus is no longer present, and therapy will not be ineffective. However, it may trigger bronchospasms [4]. The only exception for ribavirin use is in immunocompromised pediatric patients with RSV. In these patients, the virus continuously replicates for months after the initial infection in which the host defense is ineffective against this virus; therefore, aerosolized ribavirin therapy should be taken into consideration, either alone or in combination with humanized anti-RSV antibodies [4,45]. If aerosolized ribavirin is used in adults, continuous dosing (6 g over 12-18 hours daily) recommended for mechanically ventilated patients is as effective as intermittent dosing (2 g over 2-4 hours every eight hours) recommended in non-mechanically ventilated patients in preventing progression from lowering respiratory tract infections [3]. Due to its extreme cost and the many challenges of using aerosolized ribavirin, many centers have begun using oral ribavirin, although the dose and duration vary considerably [3-4]. Pediatric dosing of Ribavirin has not been well established compared to adult dosing. Moreover, ribavirin has a profile of harmful side effects. Ribavirin therapy has been studied and found to cause teratogenic, mutagenic, and carcinogenic adverse effects in animal models. Ribavirin teratogenic side effects have been observed only when it was given to rats during the first trimester of pregnancy, whereas carcinogenic effects appeared after animals were chronically fed large doses of the drug [45].

Furthermore, oral administration of ribavirin has been shown to inhibit erythropoiesis [45-46,49]. While these effects of ribavirin were noted in animal models, specifically rats, very few side effects were reported in human clinical studies [45]. The questionable list of side effects causes the administration of ribavirin to be carefully considered. Effective passive immune prophylaxis for RSV exists in the form of palivizumab, a humanized murine monoclonal antibody with activity against the RSV membrane F protein required for fusion with host cell membranes [45-46,49]. Palivizumab attaches to the F protein, neutralizing it and preventing the fusion of the virus with the epithelial cell [46]. Furthermore, palivizumab is 50 times more potent than RSV immunoglobulin (IVIG) and is administered as a 15mg/kg intramuscular injection once monthly with a recommended maximum of five doses per RSV season [4,46]. Palivizumab offers passive immunity for infants at risk for severe infection [4]. According to the most recent AAP guidelines, certain guidelines have been implemented where palivizumab prophylaxis is recommended with a maximum of five doses in the first year of life [3-4]. While five doses are typically provided to the patient, palivizumab should be continued throughout the season, if even the patient contracts RSV or outgrows the indication during the season [3,46]. Additional criteria for infants who may benefit from palivizumab include infants with neuromuscular disease and hemodynamically significant congenital heart disease [50]. It has also been noted that monthly prophylaxis should be discontinued in any child who is experiencing a breakthrough RSV hospitalization [45,49,50] The structure of the F protein is highly conserved, which makes palivizumab effective against all subtypes [29]. However, due to the relatively expensive nature of palivizumab, the administration is typically the subject of many costs and efficacy debates [45]. Palivizumab is priced based on weight-based doses for small children; therefore, the cost for an adult turns out to be exceptionally high [3]. Due to this nature, the use of palivizumab is limited to pediatric and HSCT patients [3,46].

## Conclusions

Due to the highly contagious nature of RSV, techniques to prevent transmission are of the utmost importance, especially in symptomatic individuals. Prevention techniques include frequent hand washing, sanitation, avoiding close contact with RSV-positive individuals, and covering the mouth while sneezing or coughing. Like many other viral respiratory illnesses, RSV is also self-limited in nature; however, supportive therapy including hydration, supplemental oxygen, and mechanical ventilation is required in hospitalized cases. Antivirals such as ribavirin may also be utilized. However, due to the detrimental adverse effects profile and the high costs, ribavirin is recommended for use in infants with severe illness and immunocompromised status. Immune prophylaxis may be administered; however, the use is generally reserved for infants during peak seasons. Palivizumab is used as a prophylaxis for infants who are at risk for severe infection. Due to the antigenic variation of RSV, producing a vaccine proves challenging. Inhibition of the F protein is crucial to the preparation of vaccines since it is the most conserved protein between RSVA and RSVB. Hypothetically, inhibition or neutralization of the F protein will prevent the fusion of the viral particle with the epithelial cells and, thus, prevent viral replication. While trials and studies for the appropriate vaccine and antiviral drugs are underway, the focus remains on ensuring that complications are due to a severe RSV illness, not the vaccine or its constituents.

## References

[1] Jha A, Jarvis H, Fraser C, Openshaw P (2016). Respiratory syncytial virus. SARS, MERS and other Viral Lung Infections.

[2] (2023). RSV transmission. https://www.cdc.gov/rsv/about/transmission.html.

[3] Nam HH, Ison MG (2019). Respiratory syncytial virus infection in adults. BMJ.

[4] Piedimonte G (2015). RSV infections: state of the art. Cleve Clin J Med.

[5] Collins PL, Fearns R, Graham BS (2013). Respiratory syncytial virus: virology, reverse genetics, and pathogenesis of disease. Curr Top Microbiol Immunol.

[6] Falsey AR, Walsh EE (2000). Respiratory syncytial virus infection in adults. Clin Microbiol Rev.

[7] Griffiths C, Drews SJ, Marchant DJ (2017). Respiratory syncytial virus: infection, detection, and new options for prevention and treatment. Clin Microbiol Rev.

[8] Van Royen T, Rossey I, Sedeyn K, Schepens B, Saelens X (2022). How RSV proteins join forces to overcome the host innate immune response. Viruses.

[9] Battles MB, McLellan JS (2019). Respiratory syncytial virus entry and how to block it. Nat Rev Microbiol.

[10] Thornhill EM, Verhoeven D (2020). Respiratory syncytial virus’s non-structural proteins: masters of interference. Front Cell Infect Microbiol.

[11] Liljeroos L, Krzyzaniak MA, Helenius A, Butcher SJ (2013). Architecture of respiratory syncytial virus revealed by electron cryotomography. Proc Natl Acad Sci U S A.

[12] Gomez RS, Guisle-Marsollier I, Bohmwald K, Bueno SM, Kalergis AM (2014). Respiratory syncytial virus: pathology, therapeutic drugs and prophylaxis. Immunol Lett.

[13] Bohmwald K, Espinoza JA, Rey-Jurado E (2016). Human respiratory syncytial virus: infection and pathology. Semin Respir Crit Care Med.

[14] Lu B, Ma CH, Brazas R, Jin H (2002). The major phosphorylation sites of the respiratory syncytial virus phosphoprotein are dispensable for virus replication in vitro. J Virol.

[15] Bian T, Gibbs JD, Örvell C, Imani F (2012). Respiratory syncytial virus matrix protein induces lung epithelial cell cycle arrest through a p53 dependent pathway. PLoS One.

[16] Mitra R, Baviskar P, Duncan-Decocq RR, Patel D, Oomens AG (2012). The human respiratory syncytial virus matrix protein is required for maturation of viral filaments. J Virol.

[17] Tran TL, Castagné N, Dubosclard V (2009). The respiratory syncytial virus M2-1 protein forms tetramers and interacts with RNA and P in a competitive manner. J Virol.

[18] Reimers K, Buchholz K, Werchau H (2005). Respiratory syncytial virus M2-1 protein induces the activation of nuclear factor kappa B. Virology.

[19] Collins PL, Hill MG, Cristina J, Grosfeld H (1996). Transcription elongation factor of respiratory syncytial virus, a nonsegmented negative-strand RNA virus. Proc Natl Acad Sci U S A.

[20] Cheng X, Park H, Zhou H, Jin H (2005). Overexpression of the M2-2 protein of respiratory syncytial virus inhibits viral replication. J Virol.

[21] Bukreyev A, Yang L, Fricke J, Cheng L, Ward JM, Murphy BR, Collins PL (2008). The secreted form of respiratory syncytial virus G glycoprotein helps the virus evade antibody-mediated restriction of replication by acting as an antigen decoy and through effects on Fc receptor-bearing leukocytes. J Virol.

[22] Fuentes S, Tran KC, Luthra P, Teng MN, He B (2007). Function of the respiratory syncytial virus small hydrophobic protein. J Virol.

[23] Rixon HW, Brown G, Aitken J, McDonald T, Graham S, Sugrue RJ (2004). The small hydrophobic (SH) protein accumulates within lipid-raft structures of the Golgi complex during respiratory syncytial virus infection. J Gen Virol.

[24] Carvajal JJ, Avellaneda AM, Salazar-Ardiles C, Maya JE, Kalergis AM, Lay MK (2019). Host components contributing to respiratory syncytial virus pathogenesis. Front Immunol.

[25] Chanock RM, Parrott RH, Vargosko AJ, Kapikian AZ, Knight V, Johnson KM (1962). IV. Respiratory syncytial virus. Am J Public Health Nations Health.

[26] Lambert L, Sagfors AM, Openshaw PJ, Culley FJ (2014). Immunity to RSV in early-life. Front Immunol.

[27] Law BJ, Carbonell-Estrany X, Simoes EAF (2002). An update on respiratory syncytial virus epidemiology: a developed country perspective. Respir Med.

[28] Olsen SJ, Winn AK, Budd AP (2021). Changes in influenza and other respiratory virus activity during the COVID-19 pandemic—United States, 2020-2021. MMWR Morb Mortal Wkly Rep.

[29] Black CP (2003). Systematic review of the biology and medical management of respiratory syncytial virus infection. Respir Care.

[30] Hussain A, Kaler J, Tabrez E, Tabrez S, Tabrez SS (2020). Novel COVID-19: a comprehensive review of transmission, manifestation, and pathogenesis. Cureus.

[31] Coultas JA, Smyth R, Openshaw PJ (2019). Respiratory syncytial virus (RSV): a scourge from infancy to old age. Thorax.

[32] Borchers AT, Chang C, Gershwin ME, Gershwin LJ (2013). Respiratory syncytial virus--a comprehensive review. Clin Rev Allergy Immunol.

[33] McNamara PS, Smyth RL (2002). The pathogenesis of respiratory syncytial virus disease in childhood. Br Med Bull.

[34] Branche AR, Falsey AR (2015). Respiratory syncytial virus infection in older adults: an under-recognized problem. Drugs Aging.

[35] Inamo Y, Hasegawa M, Saito K (2011). Serum vitamin D concentrations and associated severity of acute lower respiratory tract infections in Japanese hospitalized children. Pediatr Int.

[36] Haber N (2018). Respiratory syncytial virus infection in elderly adults. Med Mal Infect.

[37] Tada H, Nohara A, Kawashiri MA (2019). Monogenic, polygenic, and oligogenic familial hypercholesterolemia. Curr Opin Lipidol.

[38] (2023). Respiratory syncytial virus (RSV) disease. https://www.who.int/teams/health-product-policy-and-standards/standards-and-specifications/vaccine-standardization/respiratory-syncytial-virus-disease.

[39] Thomas RJ (2013). Particle size and pathogenicity in the respiratory tract. Virulence.

[40] Siegel JD, Rhinehart E, Jackson M, Chiarello L (2007). 2007 guideline for isolation precautions: preventing transmission of infectious agents in health care settings. Am J Infect Control.

[41] Noton SL, Fearns R (2015). Initiation and regulation of paramyxovirus transcription and replication. Virology.

[42] Vanover D, Smith DV, Blanchard EL (2017). RSV glycoprotein and genomic RNA dynamics reveal filament assembly prior to the plasma membrane. Nat Commun.

[43] Dargaville PA, South M, McDougall PN (1996). Surfactant abnormalities in infants with severe viral bronchiolitis. Arch Dis Child.

[44] Han S, Mallampalli RK (2015). The role of surfactant in lung disease and host defense against pulmonary infections. Ann Am Thorac Soc.

[45] Kneyber MC, Moll HA, de Groot R (2000). Treatment and prevention of respiratory syncytial virus infection. Eur J Pediatr.

[46] Eiland LS (2009). Respiratory syncytial virus: diagnosis, treatment and prevention. J Pediatr Pharmacol Ther.

[47] Eisenhut M (2006). Extrapulmonary manifestations of severe respiratory syncytial virus infection--a systematic review. Crit Care.

[48] Hanna S, Tibby SM, Durward A, Murdoch IA (2003). Incidence of hyponatraemia and hyponatraemic seizures in severe respiratory syncytial virus bronchiolitis. Acta Paediatr.

[49] Turner TL, Kopp BT, Paul G, Landgrave LC, Hayes D Jr, Thompson R (2014). Respiratory syncytial virus: current and emerging treatment options. Clinicoecon Outcomes Res.

[50] (2014). Updated guidance for palivizumab prophylaxis among infants and young children at increased risk of hospitalization for respiratory syncytial virus infection. Pediatrics.

